# Multidimensional color codes for chair tilings

**DOI:** 10.1107/S2053273322004065

**Published:** 2022-06-17

**Authors:** Shelomo Izhaq Ben-Abraham, Dvir Flom

**Affiliations:** a Department of Physics, Ben-Gurion University of the Negev, Beersheba, Israel; b Department of Computer Science, Ben-Gurion University of the Negev, Beersheba, Israel

**Keywords:** chair tilings, letter codes, digital codes, color codes, aperiodic structures, quasicrystals

## Abstract

Ordered aperiodic structures have been of interest to the crystallographic community for several decades, and study of them has in turn led to the study of lattice substitution systems, model sets and chair tilings. In this work a color code for chair tilings in arbitrary dimensions is presented. In two and three dimensions, it is expedient to translate the digital codes into colors. An explicit example of a three-dimensional color coding covering one octant is constructed. The tiling is then extended to the whole three-dimensional space and an indication is given of how to do this in arbitrary dimensions. Illustrations of some four-dimensional objects are also shown. The principle of color coding can be applied to other complex tilings such as brick tiling.

## Abstract 2

A color code for chair tilings in arbitrary dimensions is presented. The code can be applied also to other lattice substitution tilings.

## 1. Introduction

The interest of the crystallographic community in ordered aperiodic structures was aroused by two significant innovations, the introduction of the superspace group approach for dealing with modulated structures by Janner & Janssen (1977 ▸, 1980 ▸) and, mainly, the discovery of quasicrystals (short for quasiperiodic crystals) by Shechtman *et al.* (1984 ▸).

The work reported in this paper is part of a project generalizing one-dimensional sequences and two-dimensional structures to higher dimensions (Ben-Abraham & Quandt, 2011 ▸; Ben-Abraham *et al.*, 2013 ▸, 2014 ▸; Lee *et al.*, 2016 ▸). A preliminary short report on the present subject was published in the Proceedings of ICQ 13 (Flom & Ben-Abraham, 2017 ▸).

Lee & Moody (2001 ▸) studied lattice substitution systems and model sets and, among other things, generalized the chair tiling in principle to arbitrary dimensions. They also proved for chair tilings in all dimensions that, if each chair is marked with a single scattering point in a consistent way, for instance at the inner corner, then the set of points from the chairs of any one kind (orientation) is a model set and hence a pure-point (Bragg) diffraction set. Consequently, the diffraction spectrum of the entire tiling is pure point since it is a union of all partial spectra.

Robinson put forward a four-color code for the two-dimensional chair tiling. Here we generalize this code to arbitrary dimensions. We call the result Color Code for Chair Tiling (CCCT in what follows). Incidentally, statements about *d* dimensions are valid even in zero and one dimension, but these cases are extremely degenerate so they are of no interest. We explicitly elaborate the three-dimensional case. The two-dimensional chair tiling (sometimes also called the 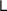 -tiling) is well known. A thorough discussion can be found in the paper by Robinson (1999 ▸), and see also Baake & Grimm (2013 ▸).

Solomyak (1997 ▸) studied the dynamics of self-similar tilings and, among others, proved that the chair tiling is pure-point diffractive. Robinson followed this with a thorough discussion of the two-dimensional chair tiling and put forward a four-color substitution tiling which codes for the two-dimensional chair tiling. A reader seriously interested in the mathematical background of the subject is advised to read these three seminal treatises. For what is to follow, we advise the reader who is not familiar with projections from higher-dimensional spaces to read *Flatland* by Abbott (1884 ▸) and consult Wikipedia on the subject (https://en.wikipedia.org/wiki/Four-dimensional_space), where numerous relevant items are presented.

## 2. Preliminaries

**Figure 1:**
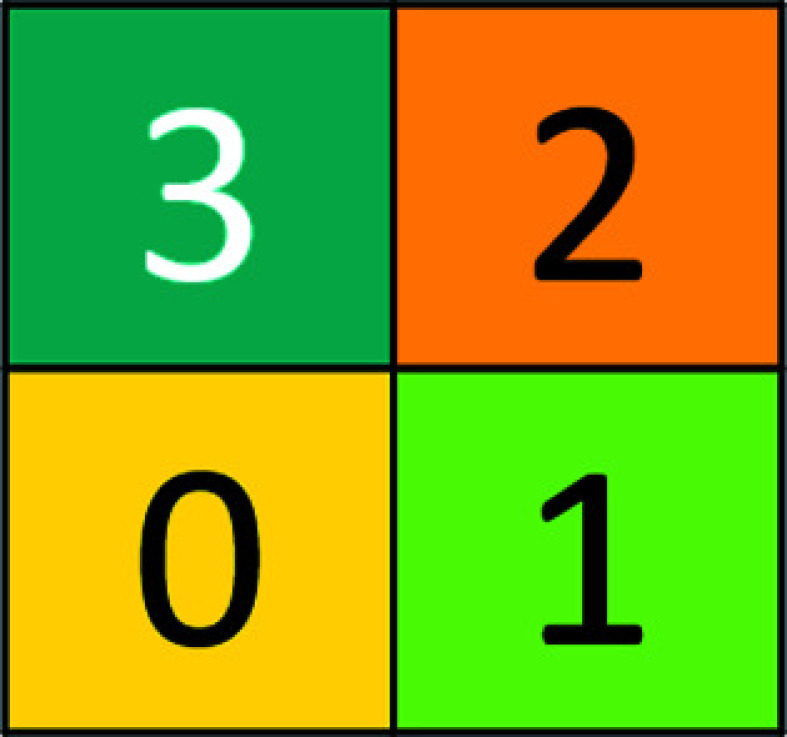
The two-dimensional basic configuration **Q**_2_.

In order to consider a *d*-dimensional chair tiling it is worthwhile starting with a basic configuration: a *d*-dimensional cube (in what follows **Q**_*d*_, or simply **Q** when there can be no confusion) with edge length of two units and composed of 2*^d^* unit cubes **q**_*d*_, or simply **q** when there can be no confusion. Figs. 1 ▸ and 2 ▸ show this for two and three dimensions, respectively. If necessary, we shall also denote by **q**(*c*) a **q** of color *c.*

**Figure 3:**
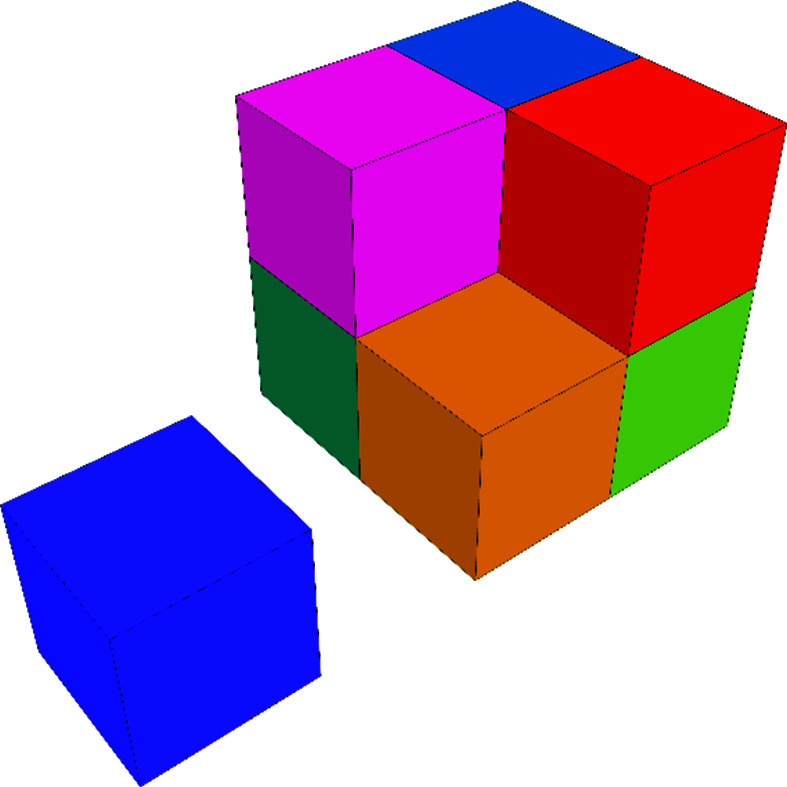
The three-dimensional chair **C**_3_ with the missing cube **q**_3_ beside it.

A *d*-dimensional chair **C** *_d_* (or simply **C** when there can be no confusion) is a **Q** *_d_* with one **q** removed. Fig. 3 ▸ shows this for three dimensions.

A chair is a rep-tile. That is to say, it can be tiled by smaller copies of itself *ad infinitum*. By the same token, it induces by inflation an infinite substitution tiling.

**Figure 4:**
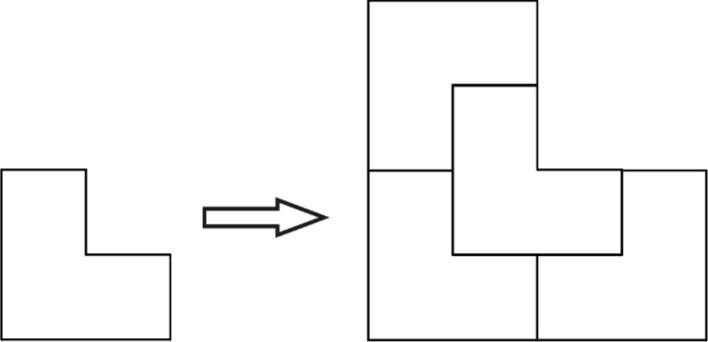
The two-dimensional chair tiling prototile and its inflation.

The original way of constructing the two-dimensional chair tiling is by inflation, and this is shown in Fig. 4 ▸.

**Figure 5:**
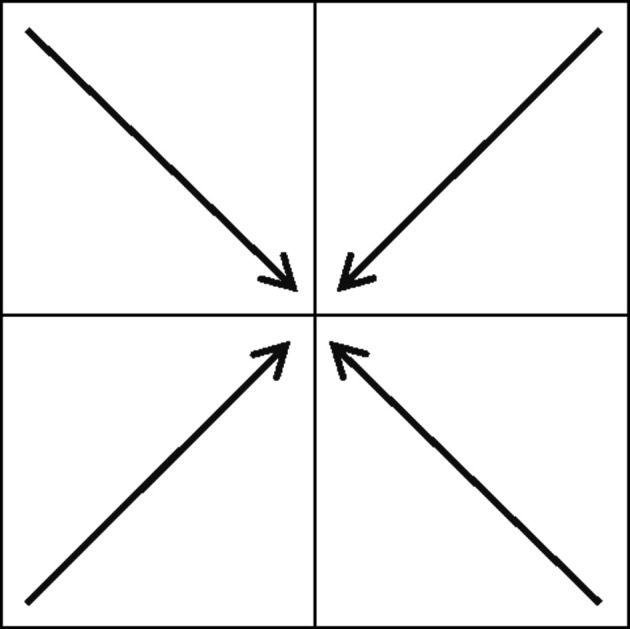
Two-dimensional labeling of unit squares **q**_2_ by arrows.

The conventional way to label the **q**’s is by arrows along one of the body diagonals, with the arrows pointing towards the center of the respective **Q**; this is shown in Fig. 5 ▸ for two dimensions. It is, however, convenient and expedient to translate the arrow labeling into an alphabet 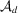 of 2*^d^* letters/digits/colors. In what follows we shall usually refer to them simply as colors. The assignment of arrows, number codes and colors for two dimensions is shown in Table 1 ▸.

**Figure 6:**
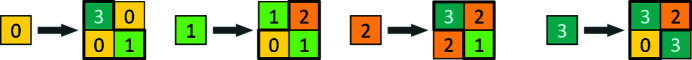
Two-dimensional chair code tiling substitution. Two-dimensional proto-chairs are drawn (shown by thick lines) and their cyclic color change is demonstrated.

**Figure 7:**
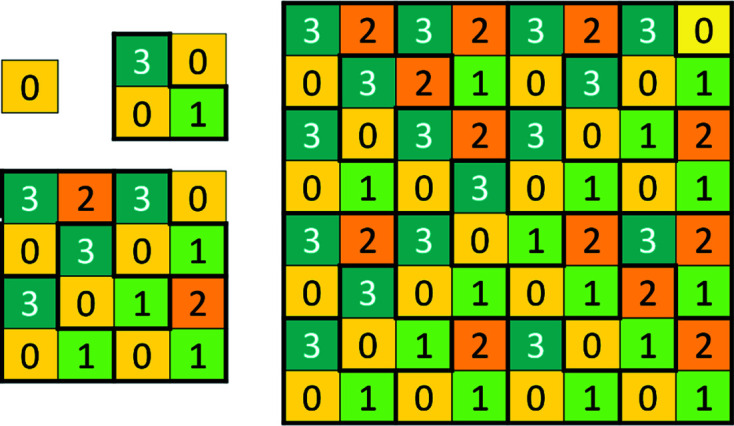
Two-dimensional CCCT. Generations 0, 1, 2, 3 are given, starting with 0, showing color-coded two-dimensional chairs.

To make this paper self-contained, and also for comparison with the construction of the two-dimensional chair tiling by inflation, we recall Robinson’s labeling by a color code and construction by substitution. The substitution is shown in Fig. 6 ▸, which also displays the two-dimensional proto-chairs (shown by thick lines) and their cyclic color change. Yet there is a caveat: the orientation of the chairs depends on the sector. What is shown is valid in the upper right quadrant [1 1]. In the lower left quadrant all chairs will be reflected (flipped) around the diagonal of unequal squares. Incidentally, we remark that while the generally accepted way of labeling the colors is by the digits 0, 1, 2, 3, Robinson originally denoted them *p*, *q*, *r*, *s*, respectively. Generations 0 to 3 of the tiling are shown in Fig. 7 ▸, where the resulting two-dimensional chairs and their inflation are also explicitly shown. We observe that the block substituting for **q**(*c*) is always a **Q**_2_, with the **q** diametrically opposite to **q**(*c*) replaced by **q**(*c*) itself.

## 3. Multidimensional color codes

**Figure 8:**
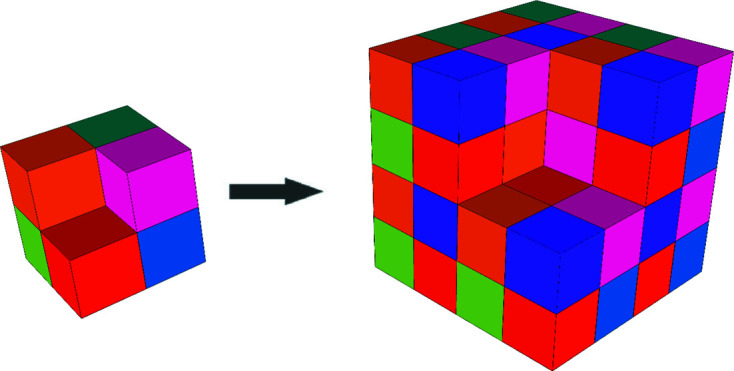
Three-dimensional chair code tiling substitution.

**Figure 9:**
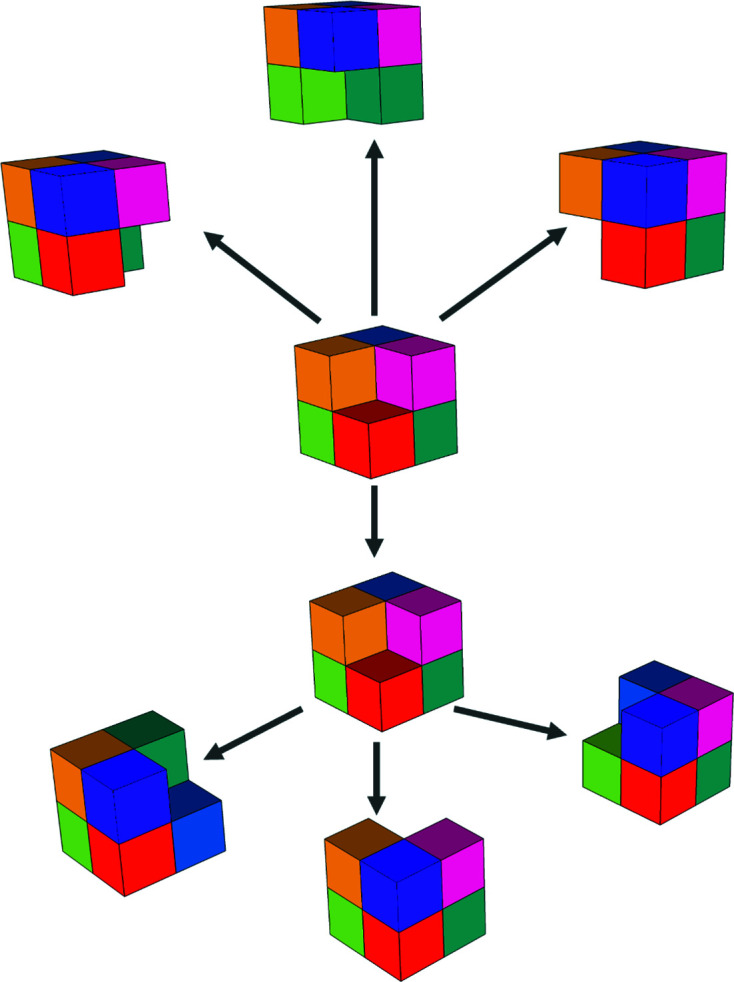
An exploded view of three-dimensional chair inflation.

For completeness, as well as for comparison with the construction by CCCT substitution, we start by showing the basics of the three-dimensional inflation (Fig. 8 ▸). An exploded view of the inflation is shown in Fig. 9 ▸.

The assignment of arrows, number codes and colors for three dimensions is shown in Table 2 ▸. The convention for three dimensions is as follows. The number codes of diametrically opposite **q**_3_’s are of opposite parity and sum to 7. This rule immediately generalizes to any dimension except 2. In higher dimensions the coding by actual colors becomes impractical. Thus, it would be probably rather hard to find 2^*d*^ sufficiently different hues for *d* ≥ 4. We observe that, within a **Q** *_d_* in any dimension, the parity of the nearest neighbors of a given **q**_*d*_ is opposite to that of **q** *_d_*. In any even dimension (such as two dimensions) the substitution upholds this rule. On the other hand, in any odd dimension (such as three dimensions) the substitution violates this rule, since the initial **q** replaces its diametrical counterpart even though their parities are opposite.

In any dimension *d*, the symmetry of the entire structure is that of a *d*-dimensional cube colored by 2^*d*^ colors. It is easy to see that the symmetry of **Q** *_d_* propagates throughout the whole structure in all generations. Thus, in two dimensions, the point symmetry group is 4′*m*′, and in three dimensions the point symmetry group is *m*′3′*m*′, where the prime indicates a change of color.

The starting **q** stays invariant throughout the main body diagonal which is the propagation direction of the structure. Consequently, its corresponding chair also propagates with it diagonally. Thus, for instance, in three dimensions starting with 0, all **q**’s in the main diagonal are 0’s (yellow) and stay surrounded by the remaining six **q**’s of the respective chair.

**Figure 10:**
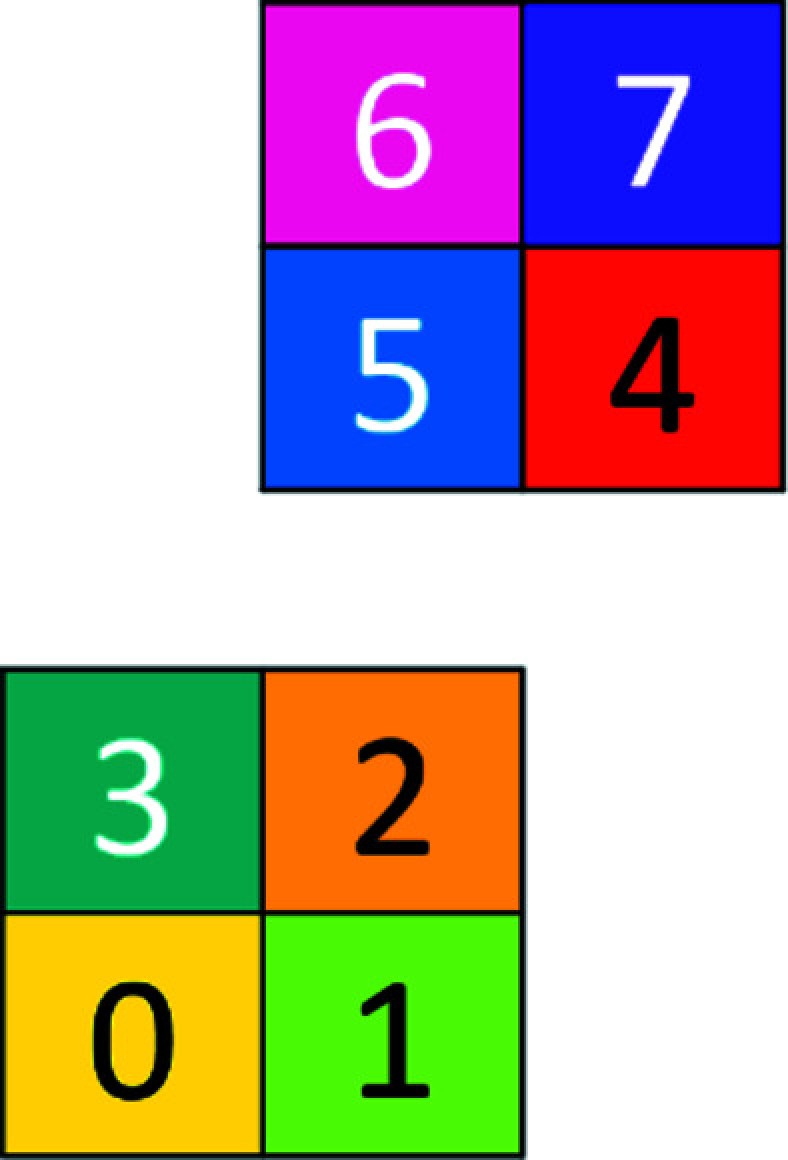
A two-dimensional graphic representation of the three-dimensional basic configuration **Q**_3_.

**Figure 11:**
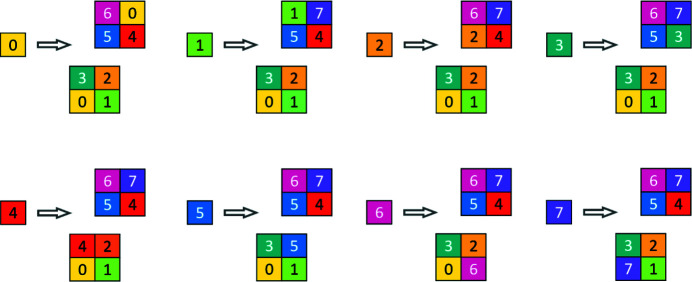
Three-dimensional chair code tiling substitution. Two-dimensional proto-chairs are drawn (shown by thick lines) and their cyclic color change is demonstrated.

**Figure 12:**
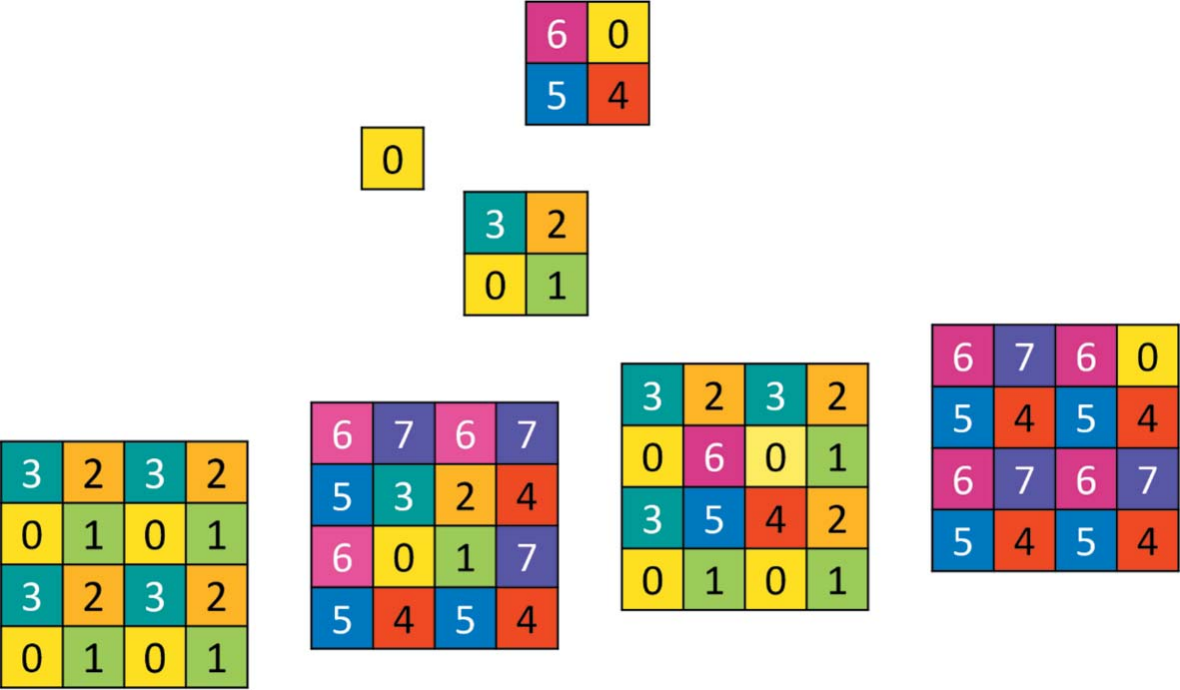
Three-dimensional CCCT, showing generations 0, 1, 2, starting with 0.

The two-dimensional code tiling substitution generalizes to arbitrary dimensions. A two-dimensional representation of **Q**_3_ is shown in Fig. 10 ▸; this is required for construction of the three-dimensional case. For three dimensions we show the substitution explicitly in Fig. 11 ▸. Generations 0, 1, 2 of the three-dimensional CCCT starting with 0 are shown in Fig. 12 ▸.

## 4. Tiling the whole space

**Figure 13:**
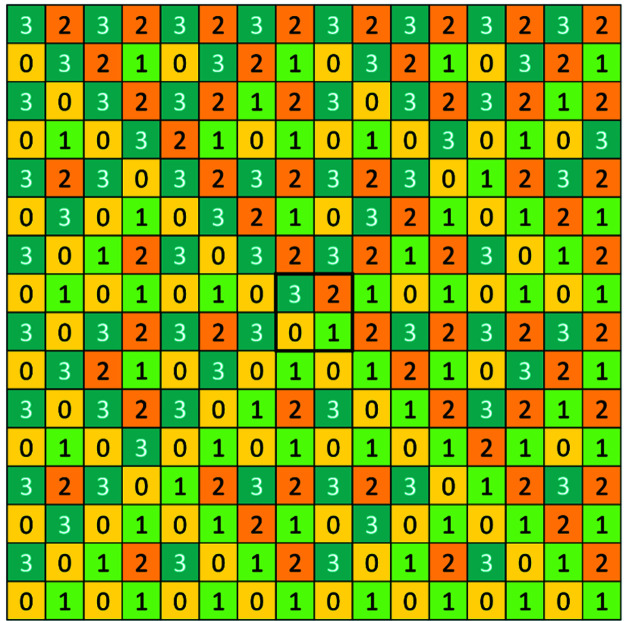
Two-dimensional CCCT: third generation starting with seeded **Q**_2_ (shown in the central frame by a thick-lined contour).

**Figure 14:**
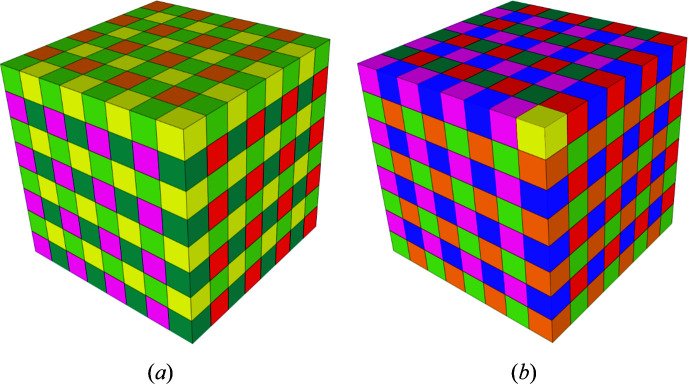
Projections of the hull of the three-dimensional second generation. (*a*) Starting vertex first, (*b*) the diametrical vertex first.

What has been said up to now refers to tilings that cover one sector (quadrant, octant *etc.*). In order to extend the tiling to the whole *d*-dimensional structure, one must start by seeding the basic configuration **Q** *_d_* and continue therefrom. For two dimensions this is shown in Fig. 13 ▸. A large two-dimensional picture can also be found in the book by Baake & Grimm (2013 ▸) even though it is in a quite different context. The diligent reader is invited to do that for three dimensions and/or look at the supporting information, which shows the sixteen 16 × 16 matrices of the third generation of the three-dimensional CCCT. Here we focus on three dimensions. Projections of the hull of the second generation of three-dimensional CCCT are shown in Fig. 14 ▸.

## 5. Four dimensions

**Figure 15:**
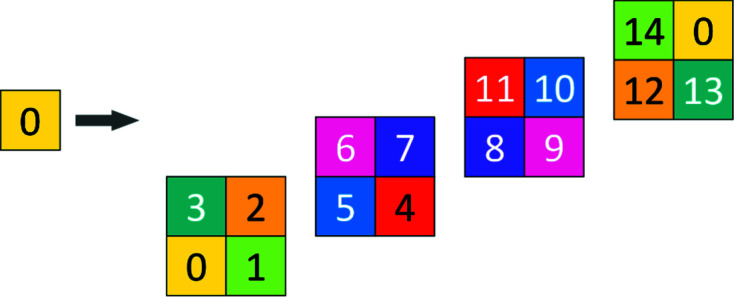
Four-dimensional CCCT: part of inflation starting with 0 (yellow).

In principle, there is no problem extending the construction to any dimension, but the requirements on space grow exponentially. Therefore, we limit ourselves to presenting only some basics. As has already been said, it is practically impossible to find 16 distinguishable hues. Therefore, to represent the four-dimensional version with colors we choose to assign the same color to diametrically opposite **q**_4_’s. Displaying the whole four-dimensional inflation would take too much space, so we show in Fig. 15 ▸ only the inflation starting with 0 (yellow).

**Figure 16:**
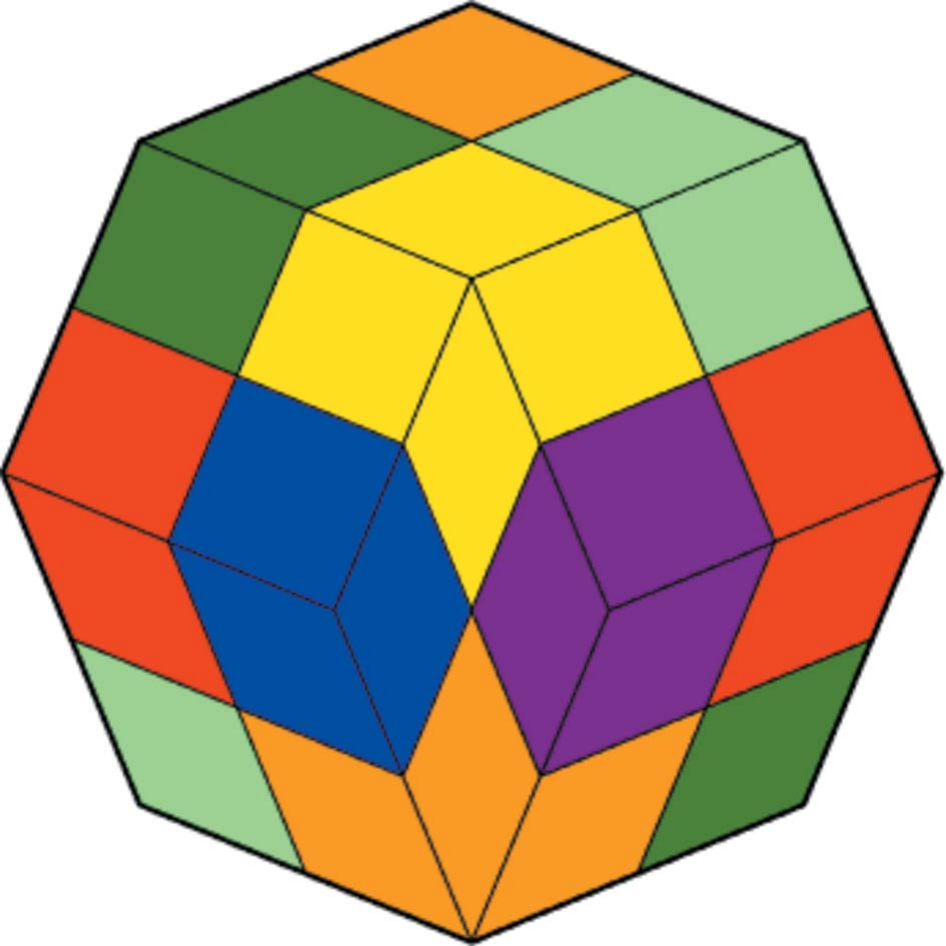
A two-dimensional projection of the four-dimensional colored basic configuration **Q**_4_.

**Figure 17:**
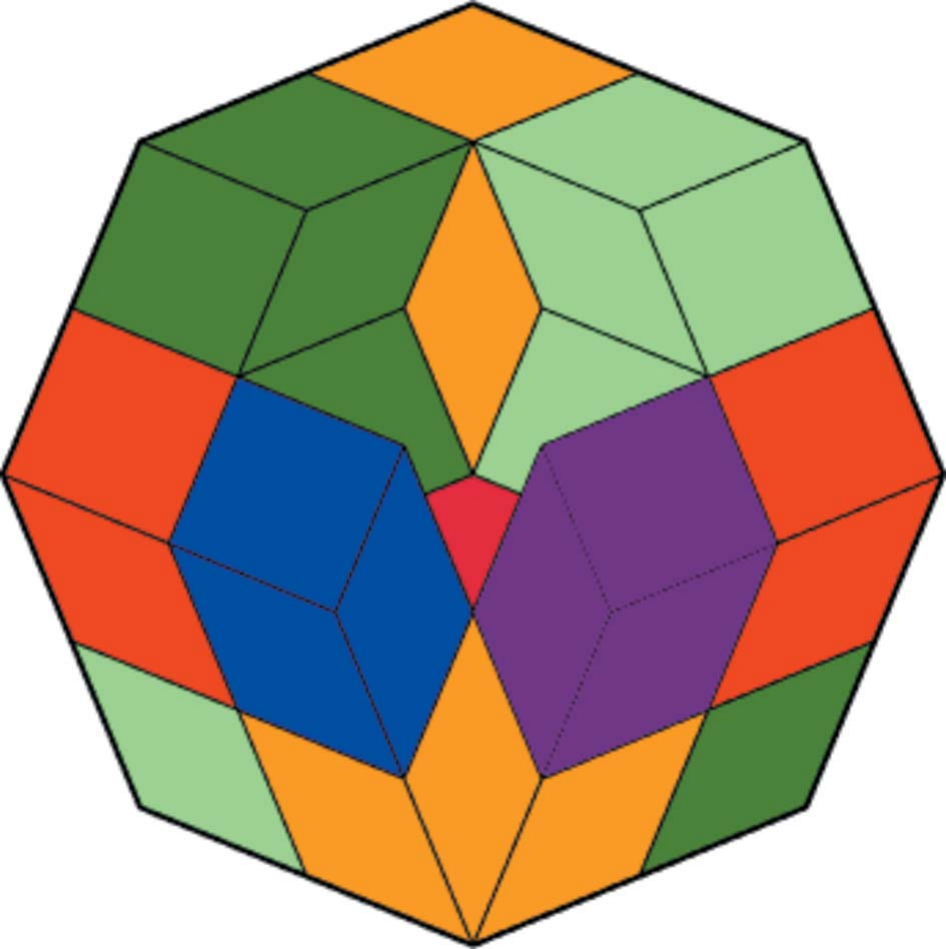
A two-dimensional projection of the four-dimensional chair **C**_4_.

**Figure 18:**
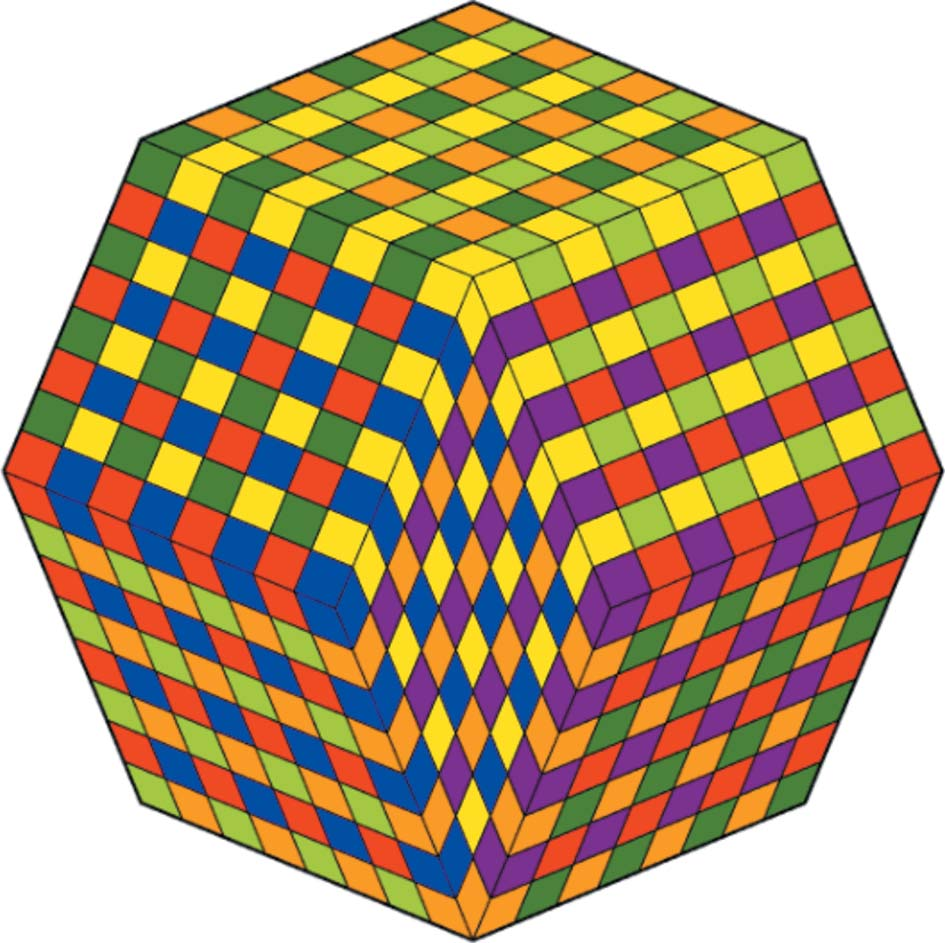
A two-dimensional projection of part of the hull of the second generation of the four-dimensional CCCT.

A two-dimensional projection of the four-dimensional colored basic configuration **Q**_4_ is shown in Fig. 16 ▸. A two-dimensional projection of the four-dimensional colored chair **C**_4_ is shown in Fig. 17 ▸. Finally, Fig. 18 ▸ shows a two-dimensional projection of part of the hull of the second generation of the four-dimensional CCCT. We remark that the isometric (short for isogonal axonometric) three-dimensional projection of a 4-cube is a rhombic dodecahedron and that, in turn, projects to two dimensions as a regular octagon partitioned as shown in the figure.

## 6. Conclusions

We have constructed a coding substitution tiling of chair tilings in arbitrary dimensions. In two and three dimensions, it is expedient to translate the digital codes into colors. We have constructed an explicit example of a three-dimensional color coding covering one octant. We have then extended the tiling to the whole three-dimensional space and indicated how to do this in arbitrary dimensions. We have also shown illustrations of some four-dimensional objects. The principle of color coding can be applied to other complex tilings such as the brick tiling.

## Supplementary Material

Figures showing the 16 matrices, each 16x16, of CCCT. Each figure is a layer of the third generation of the 3D tiling mentioned in Section 4. DOI: 10.1107/S2053273322004065/ae5109sup1.pdf

## Figures and Tables

**Figure 2:**
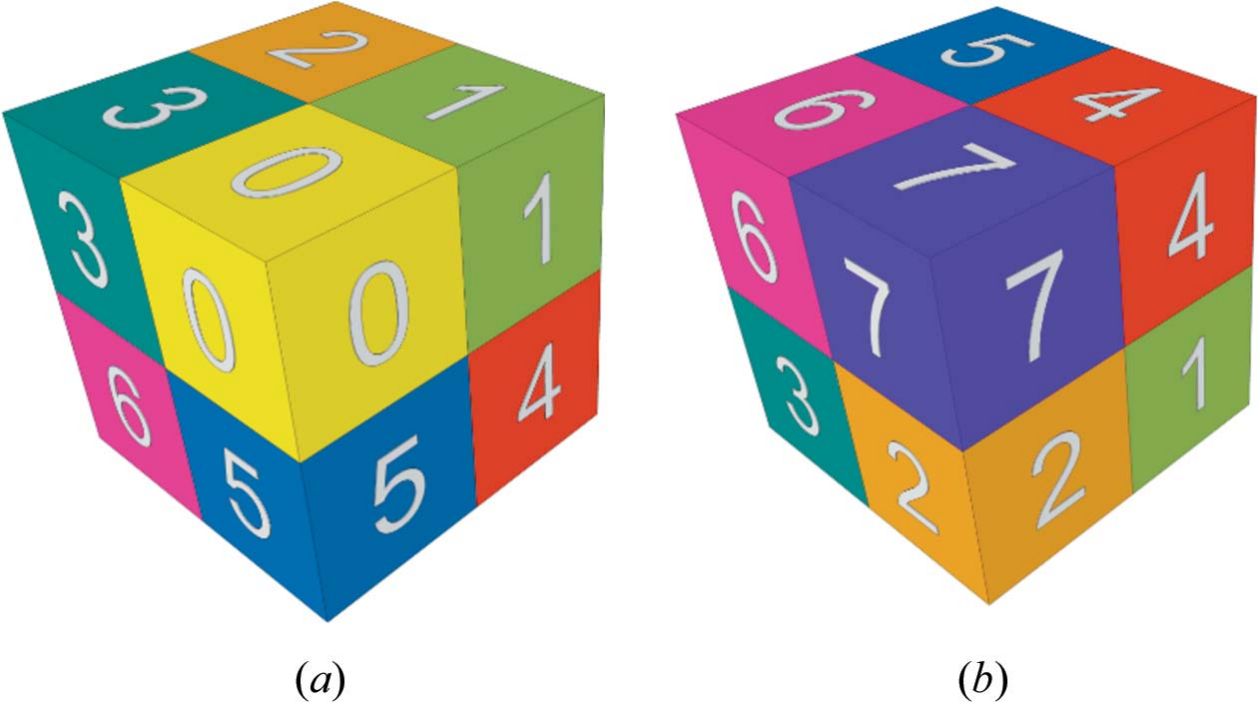
The three-dimensional basic configuration **Q**_3_. (*a*) 0 (yellow) first, (*b*) 7 (violet) first.

**Table 1:** Labeling of unit cubes **q**_2_(*c*) for two dimensions

Number code	Arrow	Color
0	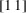	Yellow
1	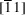	Green
2	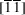	Orange
3	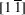	Turquoise

**Table 2:** Labeling of unit cubes **q**_3_(*c*) for three dimensions

Number code	Arrow	Color
0	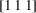	Yellow
1	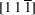	Green
2	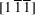	Orange
3	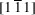	Turquoise
4	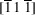	Red
5	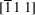	Blue
6	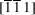	Purple
7	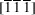	Violet
