# Supplementary material for: Multidimensional color codes for chair tilings
Source: Acta Crystallogr A Found Adv. 2022 Jun 17;78(Pt 4):359–63. doi: 10.1107/S2053273322004065 (PMC9252302; doi:10.1107/S2053273322004065)
Supplement: Supplementary file 1 [file a-78-00359-sup1.pdf]

FOUNDATIONS
ADVANCES

Volume 78 (2022)

Supporting information for article:

Multidimensional color codes for chair tilings

Shelomo Izhaq Ben-Abraham and Dvir Flom

3	2	3	2	3	2	3	2	3	2	3	2	3	2	3	2
0	1	0	1	0	1	0	1	0	1	0	1	0	1	0	1
3	2	3	2	3	2	3	2	3	2	3	2	3	2	3	2
0	1	0	1	0	1	0	1	0	1	0	1	0	1	0	1
3	2	3	2	3	2	3	2	3	2	3	2	3	2	3	2
0	1	0	1	0	1	0	1	0	1	0	1	0	1	0	1
3	2	3	2	3	2	3	2	3	2	3	2	3	2	3	2
0	1	0	1	0	1	0	1	0	1	0	1	0	1	0	1
3	2	3	2	3	2	3	2	3	2	3	2	3	2	3	2
0	1	0	1	0	1	0	1	0	1	0	1	0	1	0	1
3	2	3	2	3	2	3	2	3	2	3	2	3	2	3	2
0	1	0	1	0	1	0	1	0	1	0	1	0	1	0	1
3	2	3	2	3	2	3	2	3	2	3	2	3	2	3	2
0	1	0	1	0	1	0	1	0	1	0	1	0	1	0	1
3	2	3	2	3	2	3	2	3	2	3	2	3	2	3	2
0	1	0	1	0	1	0	1	0	1	0	1	0	1	0	1

Figure 1
CCCT 3D Gen=4 layer 1

6	7	6	7	6	7	6	7	6	7	6	7	6	7	6	7
5	3	2	4	5	3	2	4	5	3	2	4	5	3	2	4
6	0	1	7	6	0	1	7	6	0	1	7	6	0	1	7
5	4	5	4	5	4	5	4	5	4	5	4	5	4	5	4
6	7	6	7	6	7	6	7	6	7	6	7	6	7	6	7
5	3	2	4	5	3	2	4	5	3	2	4	5	3	2	4
6	0	1	7	6	0	1	7	6	0	1	7	6	0	1	7
5	4	5	4	5	4	5	4	5	4	5	4	5	4	5	4
6	7	6	7	6	7	6	7	6	7	6	7	6	7	6	7
5	3	2	4	5	3	2	4	5	3	2	4	5	3	2	4
6	0	1	7	6	0	1	7	6	0	1	7	6	0	1	7
5	4	5	4	5	4	5	4	5	4	5	4	5	4	5	4
6	7	6	7	6	7	6	7	6	7	6	7	6	7	6	7
5	3	2	4	5	3	2	4	5	3	2	4	5	3	2	4
6	0	1	7	6	0	1	7	6	0	1	7	6	0	1	7
5	4	5	4	5	4	5	4	5	4	5	4	5	4	5	4

Figure 2
CCCT 3D Gen=4 layer 2

3	2	3	2	3	2	3	2	3	2	3	2	3	2	3	2
0	6	7	1	0	6	7	1	0	6	7	1	0	6	7	1
3	5	3	2	3	2	4	2	3	5	3	2	3	2	4	2
0	1	0	1	0	1	0	1	0	1	0	1	0	1	0	1
3	2	3	2	3	2	3	2	3	2	3	2	3	2	3	2
0	6	0	1	0	1	7	1	0	6	0	1	0	1	7	1
3	5	4	2	3	5	4	2	3	5	4	2	3	5	4	2
0	1	0	1	0	1	0	1	0	1	0	1	0	1	0	1
3	2	3	2	3	2	3	2	3	2	3	2	3	2	3	2
0	6	7	1	0	6	7	1	0	6	7	1	0	6	7	1
3	5	3	2	3	2	4	2	3	5	3	2	3	2	4	2
0	1	0	1	0	1	0	1	0	1	0	1	0	1	0	1
3	2	3	2	3	2	3	2	3	2	3	2	3	2	3	2
0	6	0	1	0	1	7	1	0	6	0	1	0	1	7	1
3	5	4	2	3	5	4	2	3	5	4	2	3	5	4	2
0	1	0	1	0	1	0	1	0	1	0	1	0	1	0	1

Figure 3

CCCT 3D Gen=4 layer 3

6	7	6	7	6	7	6	7	6	7	6	7	6	7	6	7
5	4	5	4	5	4	5	4	5	4	5	4	5	4	5	4
6	7	6	7	6	7	6	7	6	7	6	7	6	7	6	7
5	4	5	3	2	4	5	4	5	4	5	3	2	4	5	4
6	7	6	0	1	7	6	7	6	7	6	0	1	7	6	7
5	4	5	4	5	4	5	4	5	4	5	4	5	4	5	4
6	7	6	7	6	7	6	7	6	7	6	7	6	7	6	7
5	4	5	4	5	4	5	4	5	4	5	4	5	4	5	4
6	7	6	7	6	7	6	7	6	7	6	7	6	7	6	7
5	4	5	4	5	4	5	4	5	4	5	4	5	4	5	4
6	7	6	7	6	7	6	7	6	7	6	7	6	7	6	7
5	4	5	3	2	4	5	4	5	4	5	3	2	4	5	4
6	7	6	0	1	7	6	7	6	7	6	0	1	7	6	7
5	4	5	4	5	4	5	4	5	4	5	4	5	4	5	4
6	7	6	7	6	7	6	7	6	7	6	7	6	7	6	7
5	4	5	4	5	4	5	4	5	4	5	4	5	4	5	4

Figure 4

CCCT 3D Gen=4 layer 4

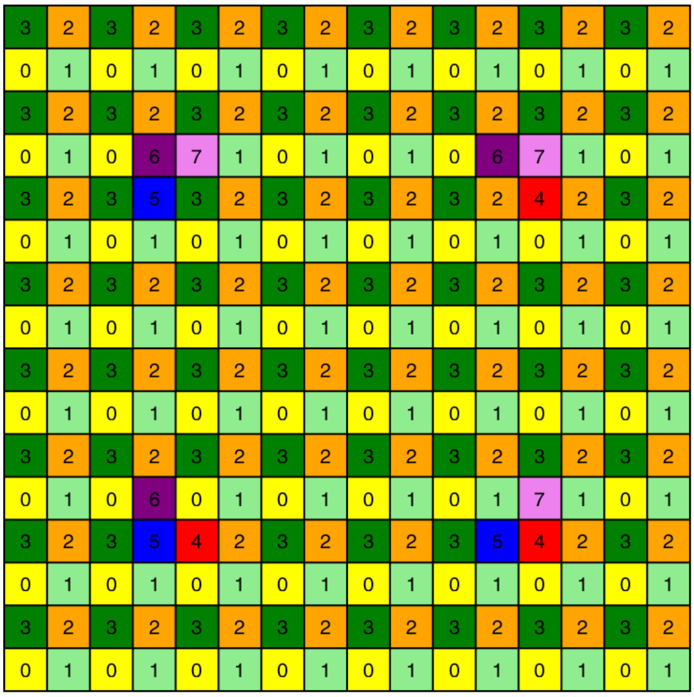

Figure 5

CCCT 3D Gen=4 layer 5

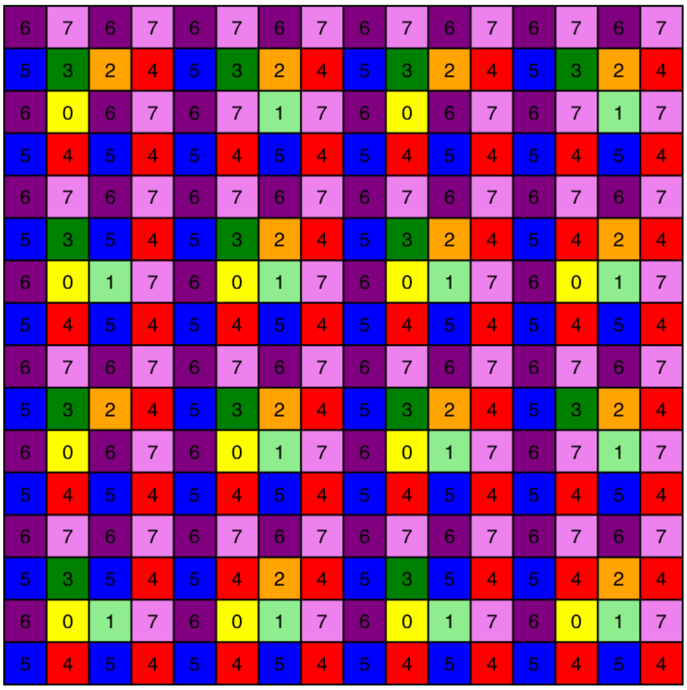

Figure 6

CCCT 3D Gen=4 layer 6

3	2	3	2	3	2	3	2	3	2	3	2	3	2	3	2
0	6	7	1	0	6	7	1	0	6	7	1	0	6	7	1
3	5	4	2	3	5	4	2	3	5	4	2	3	5	4	2
0	1	0	1	0	1	0	1	0	1	0	1	0	1	0	1
3	2	3	2	3	2	3	2	3	2	3	2	3	2	3	2
0	6	7	1	0	6	7	1	0	6	7	1	0	6	7	1
3	5	4	2	3	5	3	2	3	2	4	2	3	5	4	2
0	1	0	1	0	1	0	1	0	1	0	1	0	1	0	1
3	2	3	2	3	2	3	2	3	2	3	2	3	2	3	2
0	6	7	1	0	6	0	1	0	1	7	1	0	6	7	1
3	5	4	2	3	5	4	2	3	5	4	2	3	5	4	2
0	1	0	1	0	1	0	1	0	1	0	1	0	1	0	1
3	2	3	2	3	2	3	2	3	2	3	2	3	2	3	2
0	6	7	1	0	6	7	1	0	6	7	1	0	6	7	1
3	5	4	2	3	5	4	2	3	5	4	2	3	5	4	2
0	1	0	1	0	1	0	1	0	1	0	1	0	1	0	1

Figure 7

CCCT 3D Gen=4 layer 7

6	7	6	7	6	7	6	7	6	7	6	7	6	7	6	7
5	4	5	4	5	4	5	4	5	4	5	4	5	4	5	4
6	7	6	7	6	7	6	7	6	7	6	7	6	7	6	7
5	4	5	4	5	4	5	4	5	4	5	4	5	4	5	4
6	7	6	7	6	7	6	7	6	7	6	7	6	7	6	7
5	4	5	4	5	4	5	4	5	4	5	4	5	4	5	4
6	7	6	7	6	7	6	7	6	7	6	7	6	7	6	7
5	4	5	4	5	4	5	3	2	4	5	4	5	4	5	4
6	7	6	7	6	7	6	0	1	7	6	7	6	7	6	7
5	4	5	4	5	4	5	4	5	4	5	4	5	4	5	4
6	7	6	7	6	7	6	7	6	7	6	7	6	7	6	7
5	4	5	4	5	4	5	4	5	4	5	4	5	4	5	4
6	7	6	7	6	7	6	7	6	7	6	7	6	7	6	7
5	4	5	4	5	4	5	4	5	4	5	4	5	4	5	4
6	7	6	7	6	7	6	7	6	7	6	7	6	7	6	7
5	4	5	4	5	4	5	4	5	4	5	4	5	4	5	4

Figure 8

CCCT 3D Gen=4 layer 8

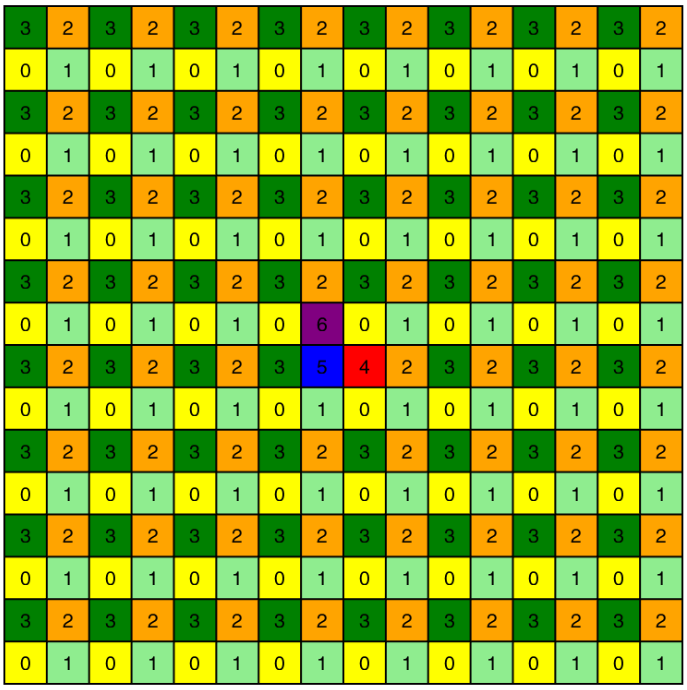

Figure 9

CCCT 3D Gen=4 layer 9

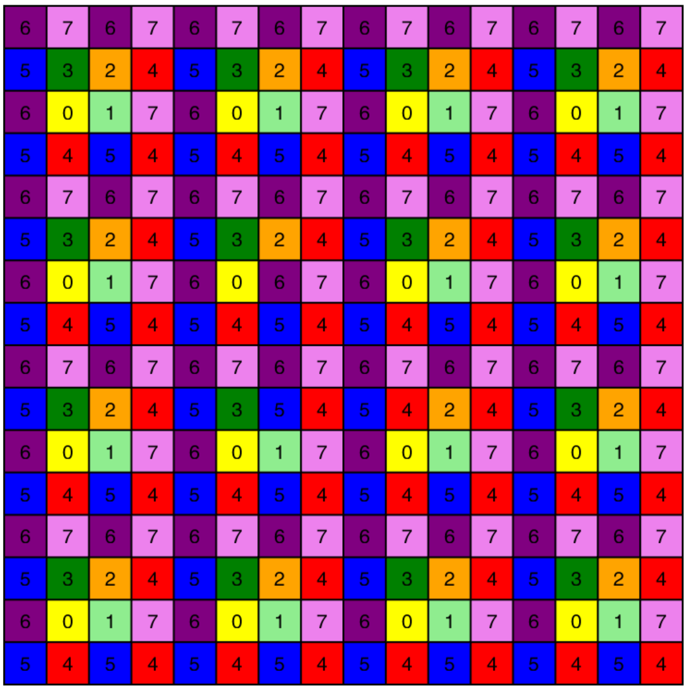

Figure 10

CCCT 3D Gen=4 layer 10

3	2	3	2	3	2	3	2	3	2	3	2	3	2	3	2
0	6	7	1	0	6	7	1	0	6	7	1	0	6	7	1
3	5	3	2	3	2	4	2	3	5	3	2	3	2	4	2
0	1	0	1	0	1	0	1	0	1	0	1	0	1	0	1
3	2	3	2	3	2	3	2	3	2	3	2	3	2	3	2
0	6	0	1	0	6	7	1	0	6	0	1	0	1	7	1
3	5	4	2	3	5	4	2	3	5	4	2	3	5	4	2
0	1	0	1	0	1	0	1	0	1	0	1	0	1	0	1
3	2	3	2	3	2	3	2	3	2	3	2	3	2	3	2
0	6	7	1	0	6	7	1	0	6	7	1	0	6	7	1
3	5	3	2	3	5	4	2	3	5	4	2	3	2	4	2
0	1	0	1	0	1	0	1	0	1	0	1	0	1	0	1
3	2	3	2	3	2	3	2	3	2	3	2	3	2	3	2
0	6	0	1	0	1	7	1	0	6	0	1	0	1	7	1
3	5	4	2	3	5	4	2	3	5	4	2	3	5	4	2
0	1	0	1	0	1	0	1	0	1	0	1	0	1	0	1

Figure 11

CCCT 3D Gen=4 layer 11

6	7	6	7	6	7	6	7	6	7	6	7	6	7	6	7
5	4	5	4	5	4	5	4	5	4	5	4	5	4	5	4
6	7	6	7	6	7	6	7	6	7	6	7	6	7	6	7
5	4	5	3	2	4	5	4	5	4	5	3	2	4	5	4
6	7	6	0	6	7	6	7	6	7	6	0	1	7	6	7
5	4	5	4	5	4	5	4	5	4	5	4	5	4	5	4
6	7	6	7	6	7	6	7	6	7	6	7	6	7	6	7
5	4	5	4	5	4	5	4	5	4	5	4	5	4	5	4
6	7	6	7	6	7	6	7	6	7	6	7	6	7	6	7
5	4	5	4	5	4	5	4	5	4	5	4	5	4	5	4
6	7	6	7	6	7	6	7	6	7	6	7	6	7	6	7
5	4	5	3	5	4	5	4	5	4	5	4	2	4	5	4
6	7	6	0	1	7	6	7	6	7	6	0	1	7	6	7
5	4	5	4	5	4	5	4	5	4	5	4	5	4	5	4
6	7	6	7	6	7	6	7	6	7	6	7	6	7	6	7
5	4	5	4	5	4	5	4	5	4	5	4	5	4	5	4

Figure 12

CCCT 3D Gen=4 layer 12

3	2	3	2	3	2	3	2	3	2	3	2	3	2	3	2
0	1	0	1	0	1	0	1	0	1	0	1	0	1	0	1
3	2	3	2	3	2	3	2	3	2	3	2	3	2	3	2
0	1	0	6	7	1	0	1	0	1	0	6	0	1	0	1
3	2	3	5	4	2	3	2	3	2	3	5	4	2	3	2
0	1	0	1	0	1	0	1	0	1	0	1	0	1	0	1
3	2	3	2	3	2	3	2	3	2	3	2	3	2	3	2
0	1	0	1	0	1	0	1	0	1	0	1	0	1	0	1
3	2	3	2	3	2	3	2	3	2	3	2	3	2	3	2
0	1	0	1	0	1	0	1	0	1	0	1	0	1	0	1
3	2	3	2	3	2	3	2	3	2	3	2	3	2	3	2
0	1	0	6	7	1	0	1	0	1	0	6	7	1	0	1
3	2	3	5	4	2	3	2	3	2	3	5	4	2	3	2
0	1	0	1	0	1	0	1	0	1	0	1	0	1	0	1
3	2	3	2	3	2	3	2	3	2	3	2	3	2	3	2
0	1	0	1	0	1	0	1	0	1	0	1	0	1	0	1

Figure 13

CCCT 3D Gen=4 layer 13

6	7	6	7	6	7	6	7	6	7	6	7	6	7	6	7
5	3	2	4	5	3	2	4	5	3	2	4	5	3	2	4
6	0	6	7	6	7	1	7	6	0	6	7	6	0	1	7
5	4	5	4	5	4	5	4	5	4	5	4	5	4	5	4
6	7	6	7	6	7	6	7	6	7	6	7	6	7	6	7
5	3	5	4	5	4	2	4	5	3	5	4	5	4	2	4
6	0	1	7	6	0	1	7	6	0	1	7	6	0	1	7
5	4	5	4	5	4	5	4	5	4	5	4	5	4	5	4
6	7	6	7	6	7	6	7	6	7	6	7	6	7	6	7
5	3	2	4	5	3	2	4	5	3	2	4	5	3	2	4
6	0	6	7	6	7	1	7	6	0	6	7	6	7	1	7
5	4	5	4	5	4	5	4	5	4	5	4	5	4	5	4
6	7	6	7	6	7	6	7	6	7	6	7	6	7	6	7
5	3	5	4	5	4	2	4	5	3	5	4	5	4	2	4
6	0	1	7	6	0	1	7	6	0	1	7	6	0	1	7
5	4	5	4	5	4	5	4	5	4	5	4	5	4	5	4

Figure 14

CCCT 3D Gen=4 layer 14

3	2	3	2	3	2	3	2	3	2	3	2	3	2	3	2
0	6	7	1	0	6	7	1	0	6	7	1	0	6	0	1
3	5	4	2	3	5	4	2	3	5	4	2	3	5	4	2
0	1	0	1	0	1	0	1	0	1	0	1	0	1	0	1
3	2	3	2	3	2	3	2	3	2	3	2	3	2	3	2
0	6	7	1	0	6	7	1	0	6	7	1	0	6	7	1
3	5	4	2	3	5	4	2	3	5	4	2	3	5	4	2
0	1	0	1	0	1	0	1	0	1	0	1	0	1	0	1
3	2	3	2	3	2	3	2	3	2	3	2	3	2	3	2
0	6	7	1	0	6	7	1	0	6	7	1	0	6	7	1
3	5	4	2	3	5	4	2	3	5	4	2	3	5	4	2
0	1	0	1	0	1	0	1	0	1	0	1	0	1	0	1
3	2	3	2	3	2	3	2	3	2	3	2	3	2	3	2
0	6	7	1	0	6	7	1	0	6	7	1	0	6	7	1
3	5	4	2	3	5	4	2	3	5	4	2	3	5	4	2
0	1	0	1	0	1	0	1	0	1	0	1	0	1	0	1

Figure 15

CCCT 3D Gen=4 layer 15

6	7	6	7	6	7	6	7	6	7	6	7	6	7	6	0
5	4	5	4	5	4	5	4	5	4	5	4	5	4	5	4
6	7	6	7	6	7	6	7	6	7	6	7	6	7	6	7
5	4	5	4	5	4	5	4	5	4	5	4	5	4	5	4
6	7	6	7	6	7	6	7	6	7	6	7	6	7	6	7
5	4	5	4	5	4	5	4	5	4	5	4	5	4	5	4
6	7	6	7	6	7	6	7	6	7	6	7	6	7	6	7
5	4	5	4	5	4	5	4	5	4	5	4	5	4	5	4
6	7	6	7	6	7	6	7	6	7	6	7	6	7	6	7
5	4	5	4	5	4	5	4	5	4	5	4	5	4	5	4
6	7	6	7	6	7	6	7	6	7	6	7	6	7	6	7
5	4	5	4	5	4	5	4	5	4	5	4	5	4	5	4
6	7	6	7	6	7	6	7	6	7	6	7	6	7	6	7
5	4	5	4	5	4	5	4	5	4	5	4	5	4	5	4
6	7	6	7	6	7	6	7	6	7	6	7	6	7	6	7
5	4	5	4	5	4	5	4	5	4	5	4	5	4	5	4

Figure 16

CCCT 3D Gen=4 layer 16
